# Supplementary material for: Are carbon emissions trading and green financial instruments synergistic? -Comprehensive quantitative research based on content analysis
Source: PLoS One. 2024 Mar 7;19(3):e0298601. doi: 10.1371/journal.pone.0298601 (PMC10919601; doi:10.1371/journal.pone.0298601)
Supplement: S1 Data — (ZIP) [file pone.0298601.s001.zip › Data/地级市Z_F工作报告.zip!/╜¡╦╒/┴1⁄4╘╞╕█╩╨/└┤╫╘╣1⁄2╓┌║┼í╛┬φ┐╦╩2╛▌═°í┐_2019.docx]

各位代表：

现在，我代表连云港市人民政府向大会作工作报告，请予审议，并请各位政协委员提出意见。

2018年工作回顾

过去一年，在市委的正确领导下，我们以习近平新时代中国特色社会主义思想为指导，全面贯彻党的十九大和十九届二中、三中全会精神，牢固树立新发展理念，紧紧围绕“高质发展、后发先至”主题主线，坚定实施“五大战略”，统筹做好改革发展稳定工作，各项事业取得全面进步。

2018年，是我市发展进程中极不平凡的一年，我们积极应对宏观形势变化，咬定目标不放松，全力以赴破难题，一批重点工程加快推进，一批重大项目落地实施，一些关系全局和长远的事项取得突破，高质发展的有利条件更加具备，后发先至的跨越势能加快聚集，连云港正以全新姿态站在新的发展起点上。

——这一年，全省重大项目现场推进会在连隆重召开，总投资775亿元的盛虹炼化一体化项目正式开工，国际先进精细化工产业加速集结，世界一流石化基地翘首可期，这也标志着我们加速新旧动能转换、推进产业强市取得重大突破，标杆示范项目打造取得重要进展，“一带一路”强支点建设迈出关键步伐。

——这一年，吞吐能力千万人次的花果山国际机场实现开工，区域性航空枢纽建设拉开帷幕；连盐、连青铁路建成通车，港城人民阔步迈进高铁时代。

——这一年，恒瑞医药参加习总书记召开的全国民营企业座谈会，中科院高效低碳燃气轮机试验装置获批建设，中复神鹰荣获国家科技进步一等奖，康缘药业荣获国家技术发明二等奖，一大批科技创新项目蓄势待发，我市在新一轮科技革命大潮中向潮而立、踏浪前行。

——这一年，我们深入践行习近平生态文明思想，率先建立政府主要负责人直管环保工作机制，全面落实中央环保督察反馈问题整改要求，以壮士断腕的勇气魄力，彻底整治“两灌”化工园区，腾挪发展空间，促进重大项目落地，走出一条经济发展和生态文明相辅相成、相得益彰的“连云港路子”。

——这一年，我们坚持“刀刃向内”，深化“放管服”改革，推进国家东中西区域合作示范区相关政策落地，徐圩新区获得部分省级投资管理事项权限。在全省率先开展“强区放权、市区同权”改革，委托下放权力事项3395项，真正把指挥权交给一线“听得见炮声的人”。

——这一年，我们以空前力度推进旧城改造，棚改新开工2.6万套，征收房屋590万平方米，努力解决群众住房难的问题。我们带着感情抓信访，扎实细致化矛盾，进京非访、集访分别下降76%和69%，一大批与群众生活息息相关的急事难事得到有效解决。广大公务人员深入一线，务实工作，公开公正处理问题，倾力倾情服务群众，形成干群一心、同向发力的良好局面。

——这一年，让我们尤为感动的是，习总书记对“时代楷模”王继才先进事迹作出重要指示，孤岛夫妻哨32年的坚守感动中国，爱国奉献、爱岗敬业的精神激励着港城人民撸起袖子加油干，以不懈奋斗的精神书写新时代的“西游记”。

过去一年，全市预计实现地区生产总值2800亿元，增长5%左右；一般公共预算收入234.3亿元，增长9.1%，增幅位居全省第三；固定资产投资1860亿元，增长8%；社会消费品零售总额1132亿元，增长8.5%；城乡居民人均可支配收入均增长9%。居民消费价格指数、节能减排完成省定目标。50件民生实事基本完成。受多重因素影响，个别指标没有达到预期，但仍处在合理区间。

一年来，我们主要做了以下工作：

（一）致力高质发展，抓投入，调结构，经济运行稳中向好。坚定不移推进工业立市、产业强市，千亿产业投资年度目标基本实现，新建项目开工率和竣工率均创历史最好水平。财政收入质量提高，税收占比80%。单位GDP能耗下降3%、建设用地占用下降6.4%。高能耗产业增加值下降8.9%。工业经济快速发展，应税销售收入2676亿元，增长13.6%，28家优强企业增长23.4%。中化循环经济产业园建设全面启动，新海石化、卫星石化、东华能源等加快推进。中复神鹰T1000碳纤维、正崴新能源正式投产，田湾核电3、4号机组投入运营。中复连众、天明机械成为国家制造业单项冠军示范企业。现代服务业加快集聚，连云港入选国家物流枢纽布局承载城市，大伊山、连岛获批省级旅游度假区，全市游客量和旅游收入分别增长12%和15%。新增金融机构4家，社会融资规模增量600亿元。网络零售增长30%，快递业务增长44%。农业现代化稳步推进，粮食丰产丰收，高标准农田、高效设施农业占比分别达到59%和20%，农业机械化综合水平达82%。国家农业开放合作试验区启动建设，农产品出口超6亿美元。推进国家农产品质量安全市创建，“连天下”品牌影响力扩大。获批全国海洋经济发展示范区，农发集团获评国家级农业龙头企业。自主培育海水稻“连鉴5号”亩产近千斤。与中科院共建育种研究中心。非洲猪瘟疫情有效防控。

（二）致力开放发展，谋合作，树品牌，标杆示范项目有力推进。重大基础设施不断完善，30万吨级航道二期工程加快建设，徐圩港区防波堤全面建成，旗台作业区专用铁路试运行。铁路综合客运枢纽一期投用，连镇、连徐高铁快速推进。连淮、连宿高速加快实施，204、268等国省干线改造通车。“两基地”建设强力推进，中哈物流基地集装箱进出量增长10%，哈国东门无水港过境箱量突破10万标箱。上合物流园获批国家示范园，物流量和营收分别增长65.7%、53.7%。“大通道”功能显著提升，港口吞吐量2.36亿吨，集装箱运量473万标箱。新开至南非等集装箱航线6条，内河航线苏北全覆盖，海河联运突破千万吨。国际班列开行827列，东行货运量增长60%以上。机场旅客、货物吞吐量分别增长41%、80%。开放型经济结构优化，实际利用外资6亿美元，其中战略性新兴产业、现代服务业分别占比29.5%、24.2%，“一带一路”沿线国家投资占比20.7%。外贸进出口90亿美元，增长10%。国家级综合保税区获批，连云港口岸赣榆港区、徐圩港区和灌河港区获批开放。市开发区、东海县分别获批国家级新医药、新材料出口基地。灌南经济开发区、东海高新区升格为省级开发区。成功举办连博会、连云港论坛、农洽会和国际医药技术大会，在谈签约项目增长39.6%。

（三）致力创新发展，增动力，添活力，后发优势加快培植。10个重点领域改革统筹推进，68项市级以上重点改革扎实开展。深化供给侧改革，淘汰整合冶金及化工企业18家、化解船舶产能30万载重吨，商品住宅去化周期控制在合理范围。130个补短板项目加快实施。“放管服”改革成效明显，政务服务实现镇村全覆盖，90%以上业务网上办理，企业开办登记平均用时1.5天。贸易便利化水平提升，口岸提效降费取得实效。税收征管体制改革落地实施，行政执法三项制度试点、省经济发达镇行政管理体制改革稳步推进。公立医院综合改革全省领先，文化、教育等改革取得新进展。国企改革纵深推进，20户僵尸企业整合盘活，10户企业完成混改，“三供一业”分离移交基本完成。农村集体产权制度改革扎实开展，国有林场改革通过省级验收。创新驱动战略深入实施，国家创新型城市、国家知识产权试点城市建设稳步推进，研发投入占比1.92%，国家专利优秀奖数量苏北第一。新增国家高新技术企业37家。城市政务大数据中心投用。恒瑞、天晴、康缘位居中国创新力药企前三甲，新药创制获批国家科技重大专项16项。22个项目入围国家、省级科学技术奖。新引进“千人计划”专家6人，入选省“双创计划”人才47名。30万吨级航道一期工程荣获中国土木工程詹天佑奖。

（四）致力绿色发展，下决心，出实招，生态文明建设成效显著。中央环保督察“回头看”交办事项全部办结。化工园区环保设施提档升级，关停取缔企业75家。环境综合治理全面推进，“263”专项行动扎实开展，全年空气优良率达77.3%，位居全省前列。落实河长制、湾长制、断面长制，地表水国省考断面全面消除劣V类，入海河流水质明显改善，近岸海域水质优良率达91.7%，县级以上水源地环境问题整治完成。加强生态保护修复，强化土地利用计划管理，严格落实“三线一单”，全省率先实施海洋生态红线保护规划，陆域生态红线保护面积占比达22.3%。开展国家生态文明建设示范市、国家森林城市创建，新增造林20万亩，林木覆盖率达28.3%。加强湿地保护，整治修复海域海岛岸线6公里，国家和省级生态乡镇覆盖率分别达25%、85%。花果山国家地质公园正式命名。

（五）致力协调发展，优布局，强功能，城乡融合得到新加强。城市组团加快发展，赣榆加速融入主城区，海州老城区加快改造更新，连云新城规划开发全面提速。县城发展能级稳步提升。242省道朝阳互通、环云台山大道等联通工程全面建成。城市配套不断完善，市区新改建主次干道50公里，新增供水、燃气、污水管道125公里。改造供电线路2211公里。调整优化公交线路11条，新增智慧站点100个。新开工海绵城市项目7个、地下综合管廊15.6公里。瀛洲公园、人文纪念园开工建设，新增公共绿地270公顷。国内首条生活垃圾干化生产线投用，162个小区实现垃圾分类。居住条件持续改善，整治危旧房5.2万平方米，改造老旧小区51个。开展既有住宅加装电梯试点。公租房分配率98.6%。存量房交易“四价合一”做法全国推广。农村基础设施得到加强，新改建农村公路810公里、桥梁30座。改造供水管网212公里，新增受益人口12.8万。农村改厕7.2万座。新增14个乡镇开通镇村公交。石梁河镇树墩村等8个省级美丽乡村、李集镇新民村等5个省级特色田园乡村加快建设。

（六）致力共享发展，办实事，惠民生，群众生活一年更比一年好。就业创业富民增收行动稳步实施，城镇新增就业6.3万人，自主创业1.5万人，转移农村劳动力2.5万人。新登记市场主体8.4万户，总数突破40万户。教育现代化保持苏北领先，新建中小学、幼儿园42所，改造中小学校舍45.9万平方米，71所学校试行“弹性离校”，普高扩招11%。校外培训机构办学进一步规范。健康城市建设有序推进，国家卫生县城和省级卫生乡镇实现全覆盖，医联体建设惠及基层百姓，灌云、赣榆创成全省基层卫生十强县区。“健康宝贝”工程全面实施，出生缺陷防治工作走在全国前列。首届全民健身运动会成功举办。中国排球博物馆建成开馆。红十字应急救护技能竞赛获省一等奖。全国文明城市创建深入开展。中国好人榜10月发布仪式在连举行。赣榆新乡贤文化建设获评全国宣传思想文化创新奖。《连云港年鉴（2017）》获全国特等奖。推进历史文化名城申建。建成420家基层综合性文化中心，开展文化惠民活动2万场次。8家公共图书馆获评国家一级馆，居民综合阅读率苏北第一。成功举办中国（连云港）与中亚五国“丝路迎新年”晚会。社会保障水平持续提升，医保报销比例稳步提高，医疗救助比例达75%。农村低保标准提高到480元/月以上，残疾人“两项补贴”惠及5.8万人，残疾儿童康复机构规范化建设全国推广。每千名老人拥有养老床位38.1张。对8类老人提供助餐、助浴等20多项服务补贴。发放助学金、助学贷款2.7亿元。廉租房货币补贴1048户，发放价格补贴1440万元。改造1801户贫困户危房，5.4万低收入人口和53个省定薄弱村实现脱贫。推进“最安全城市”建设，扫黑除恶斗争深入开展，群众安全感提升至96.1%。“一委三会”和社区网格治理实现全覆盖，建成国家级“民主法治示范村（社区）”　10个，法治连云港建设得到加强。诚信连云港建设持续深化。安全生产形势总体平稳。质量强市、食品药品监管扎实推进。非标三四轮车整治取得实效。殡葬改革和散葬乱埋治理成效明显。支持国防和军队建设，加强民族宗教、外事侨务、机关事务、12345在线、地震气象、邮政通讯等工作，审计、统计、民防、仲裁、档案、援疆、援外医疗等工作取得新成绩，妇女、儿童、青少年、老龄、慈善、职工维权、关心下一代、哲学社会科学等工作取得新发展。

过去一年，我们深入推进“五型”政府建设，持续转职能，改作风，提效能。修订政府工作规则，完善重大事项决策机制，坚持依法行政，推进政务公开，召开新闻发布会130场。自觉接受人大法律监督、工作监督和政协民主监督，办理人大代表议案建议221件、政协委员提案408件，办复率100%，满意率99.7%。

各位代表，成绩取得来之不易，这是省委、省政府和市委坚强领导、科学决策的结果，是全市人民团结奋斗、顽强拼搏的结果。在此，我代表市人民政府，向全市人民，向各位人大代表、政协委员，向各民主党派、工商联、人民团体、社会各界人士和离退休老同志，向驻连部队、武警官兵、公安干警，向为连云港发展作出贡献的驻连部省属单位，向所有关心支持连云港的海内外朋友，表示衷心感谢和崇高敬意！

在总结成绩同时，我们也清醒认识到：发展不充分仍是主要矛盾，新旧动能转换和产业升级步伐还不够快；城乡融合发展仍需持续发力，城市精细化管理水平有待提升；营商环境亟需大力改善，投资创业和企业经营还存在一些堵点痛点；优质服务供给仍然不足，群众在就业、看病、养老、出行等方面还存在一些困难，农村人居环境特别是在饮水安全、集中居住及环境卫生等方面还需改善；政府效能有待进一步提高，一些公务人员作风不实、缺乏担当、不作为等问题不同程度存在。对此，我们将高度重视，采取扎实措施，着力加以解决。

2019年主要任务

今年是新中国成立70周年，是全面建成小康社会的关键之年，也是实施“高质发展、后发先至”三年行动计划的大突破之年。做好今年工作，意义重大。

今年政府工作总体要求是：以习近平新时代中国特色社会主义思想为指导，全面贯彻党的十九大和十九届二中、三中全会精神，按照“五个坚持”“六个稳”工作要求，深入实施“五大战略”，坚决打好“三大攻坚战”，扎实推进经济转型、城市转型、社会转型，加快打造“一带一路”强支点，全力夺取“高质发展、后发先至”新胜利，为高水平全面建成小康社会收官打下决定性基础。

全市经济社会发展主要预期目标是：地区生产总值力争增长7%；一般公共预算收入增长8%，力争实现两位数增长；实际利用外资力争突破7亿美元；研发投入占比力争达到2%；固定资产投资、社会消费品零售总额和全体居民可支配收入均增长8%；空气优良天数保持全省领先，化工园区整治通过省级验收；居民消费价格指数、节能减排完成省定目标。

围绕市委确定的“八大突破”，重点抓好以下工作：

（一）深化改革扩大开放，加快集聚高质发展新动能。当前，“一带一路”倡议引领的新一轮经济全球化加快形成，作为国家首批沿海开放城市、“一带一路”支点城市，连云港更应以“踏平坎坷成大道”的奋斗精神，再扬开放风帆，矢志改革创新，勇立时代潮头。

全方位高水平扩大开放。发挥新亚欧大陆桥东方起点和中西部最便捷出海通道的优势，深入推进经贸、旅游、科技、文化等国际交流合作。扩大向东开放，打好“徐福”牌，以日韩、东盟为主要目标，密切经贸往来，探索建立日韩中转物流基地，进一步提升合作水平，携手打造利益共同体。加快向西开放，拓展“西游”路，以上合组织国家为重点，完善陆桥物流节点布局，持续深化通关合作，精心打造国际班列品牌，扩大两基地、出海口功能辐射面，拓展产能和商贸合作。构筑开放新平台，完善国际贸易“单一窗口”功能，综合保税区封关运作。集成创新自贸区试点经验，推进新一轮改革开放连云港“试验田”建设。积极申报自由贸易试验区。创新收益共享机制，将国家东中西区域合作示范区真正建成中西部地区的出海口和进出口产品加工基地。强力推进招商引资，锻造一支有“三头六臂”“七十二变”本领的专业招商队伍，瞄准重点地区、重点行业、重点企业，扎实开展产业链招商、精准招商、以商引商，加快招引基地型、龙头型、标志性重大项目。市开发区努力打造现代开发园区的“升级版”，徐圩新区加快形成石化产业新高地，高新区重点培育新兴产业增长极。各类开发园区明确主攻方向，加快形成千亿级、百亿级的“雁阵”梯队。大力拓展对外贸易，推广跨境电商“两平台、六体系”做法，推进货物贸易和服务贸易快速发展。全面整合展会资源，精心组织“连云港博览会”系列活动，打造“一带一路”交流合作高端平台。

宽领域深层次推进改革。坚持向改革要动力，精准推进供给侧改革，加强房地产市场监管，优化企业债务结构，落实降成本各项政策，继续实施一批补短板项目。进一步增强企业能动性，提升产业链水平，推行公平竞争审查，畅通经济循环。结合市县机构改革，深化交通、城管、文化等综合执法改革，抓好财税、金融、价格体制改革，稳步推进事业单位分类改革。坚持放到位、管到底、优服务，深入推进“放管服”改革，全面推行“双随机、一公开”监管模式，建立以信用承诺为基础的新型监管机制。推进政务服务标准化建设，实现“马上办、网上办、就近办、不见面办”，全面提升“一网通办”水平。加强国资国企改革，健全现代企业制度，提高管理水平，理顺体制，压缩层级，做强主业。推行PPP等模式，加快公共服务领域市场化改革。基本完成农村集体产权制度改革，深化集体林权制度改革，开展农业水价综合改革，重视农业保险和信贷担保体系建设，推行农田托管，进一步激活农业农村发展活力。

多主体大力度推动创新。建设重大创新载体，推进中科院大科学装置项目，规划燃气轮机产业园。增强企业创新能力，新增国家级高新技术企业30家、企业研发机构20个以上，大中型工业企业和规上高新技术企业85%以上建有研发机构。开展“三千”技改行动，实施重点项目328个，建设智能制造项目65个，新认定中小型科技企业100家。万人发明专利拥有量7件。提升科技服务水平，落实企业研发扶助政策，强化知识产权保护运用，推动众创空间发展。引导更多资本投向新兴产业和初创企业，发放“苏科贷”2亿元。新增省级以上两化融合贯标试点企业2家，引进培养高层次人才3000人、高技能人才4000人。

（二）突出工业立市产业强市，加快壮大实体经济。紧抓产业发展这个“牛鼻子”，毫不松懈抓项目，持之以恒抓投入，加快打造有区域影响力的重点产业中心，产业投资力争完成1300亿元，工业应税销售收入增长20%。

全力突破临港产业。按照四个“世界一流”标准，高水平建设石化产业基地，重点抓好盛虹炼化、东华能源、中化国际、卫星石化等重大项目建设。推动石化产业链式发展，力争再开工一批千亿级项目。大力开展绿色制造、装备升级等行动，加快推动冶金、化工等行业转型升级。积极争取国家布点建设重点能源港，推动燃气、石油等能源产业发展。加快国家海洋经济发展示范区建设，积极培育海洋生物医药、海洋可再生能源、海洋高端装备等战略性新兴产业。

做大做强优势产业。规划建设生物医药产业园，推进恒瑞、天晴、康缘、豪森等重点项目，建设省级现代中药制造业创新中心，申报国家医药创新中心，争创国家原料药生产基地，全力打造“中华药港”。发展壮大东海硅材料产业基地，加快建设中复神鹰第二基地，美景荣生物基材料建成投产，推动联瑞新材料等企业上市，争创新材料国家技术创新中心。推动核电5、6号机组扩建，延伸核电、风电、光伏发电产业链，加快发展新能源产业。鼓励自主研发成套装备，推进大型矿产机械、智能机械、港口机械等专用产品生产，打造全国重要的高端装备制造业基地。培育发展信息产业，高新区创建省级软件园，推动与陆桥沿线物流节点数据交换，建设连云新城“一带一路”交汇点大数据产业园。

提升发展特色产业。把握高铁开通机遇，加快建设国家旅游改革创新先行区和全域旅游示范区，深度整合旅游资源，精心打造大花果山景区，开展连岛、东海水晶城5A景区创建，推动文化旅游融合发展，吸引更多人来“连”做客、留“连”忘返。积极推动大健康与大生态、大旅游深度融合，引入社会资本，加快培育健康养老、海滨疗养、中医药养生、健身休闲运动等健康产业。大力发展西游动漫、电子竞技、影视出版等文化创意产业。加快发展邮轮经济、会展经济，扶持发展电子商务、智慧物流。

大力发展民营经济。落实各项政策措施，切实解决民营企业不愿投、不能投、不敢投、不知道往哪里投等问题。加大民营企业和小微企业的支持力度，建立健全中小企业服务平台，设立中小企业发展资金，放大政府性投资基金效应，组织各类投融资对接活动，提高金融服务可及性，切实解决融资难融资贵问题。对新兴产业监管坚持包容审慎，鼓励民营企业加强模式、业态、品牌创新发展。构建“亲”“清”政商关系，做到敢“亲”、真“亲”，又品“清”、行“清”，围着企业转，跟着项目跑，多与企业家交朋友，积极帮助解决难题，推动民营企业做大做强。

（三）完善重大基础设施，加快打造“一带一路”强支点。抓住我市成为国家物流枢纽机遇，进一步加强基础设施建设，统筹推进港航路园，完善口岸服务功能，发挥在全国物流网络中关键节点、重要平台和骨干枢纽的作用，助力“一带一路”标杆示范项目建设。

强化港口基础支撑。按照“枢纽港、产业港、物流港、贸易港”定位，加快建设东方大港。30万吨级航道二期建成连云港区段，启动徐圩港区段，推进铁路多式联运中心建设。加快建设盛虹炼化30万吨级码头和卫星石化等配套码头，完成赣榆港区LNG码头前期工作。推进液体散货区管廊及公共配套工程。“两翼”港口铁路专用线加快前期工作。大力提升航运能力，优化航线布局，新辟集装箱航线5条以上，港口吞吐量继续保持增长。

加快“两基地”提档升级。中哈物流基地优化作业流程，提升自动化生产水平，集装箱进出量突破30万标箱。深化与哈国铁、中远海运合作，继续推进东门无水港建设，力争完成换装量15万标箱。与沿线国家口岸建立合作机制，共建装卸场站、分拨中心、海外仓等载体，打通全程物流通道。上合物流园加快专用铁路等项目建设，物流量突破1500万吨。强化国际班列品牌，加强西向货物开发，稳定中亚东向货源，实现班列重去重回。

构建立体交通网络。开通连淮铁路，加快建设连徐高铁，推进综合客运枢纽二期工程。开展沿海高铁、连临高铁前期工作。加快连宿、连淮高速新扩建，实施311、242等国省干线改造。推进连宿徐航道启动建设。新机场土建工程基本完工。力争开通大阪、仁川等国际航线，全年旅客吞吐量突破200万人次。合理安排车船运力、班次航线，推动海陆空铁集成发展，为连云港发展装上“风火轮”。

（四）强化功能品质提升，加快“四个城市”建设。立足山海相拥、港城一体的特色优势，坚持大手笔规划、高标准建设、人性化管理，让我们的城市家园真正靓起来，动起来，活起来。

建设特色海滨城市。坚定推进城市东进，调整优化规划设计，打造百里黄金海岸。连云新城加快建设现代化、国际化、智能化、生态化城市新中心，打造“一带一路”交汇点“外滩”。老港区加快退港还城，做美滨海“客厅”。赣榆城区深度融合，拓展发展空间。海州城区大力推进旧城改造，加快有机更新，展示中心城区全新形象。推进各区之间、高铁枢纽至新机场等快速通道建设，开通市郊列车，加快形成特色彰显、功能互补、快速联通、组团发展的城市格局。

建设美丽公园城市。注重生态建设和功能集成，促进“人城境业”和谐发展。继续开展国家森林城市创建，加强城市公园建设，推进园博园等重大项目，开工建设刘志洲山体育公园、河滨公园等8个大型公园，临洪河口湿地公园建成开放。强化可进入、可参与，打造城市绿道、城市绿岛、公园社区、公园街区，推进共建共享，让市民可亲可触。突出景观化、精致化，提升改造关键节点、重要道路、重点空间绿化美化，建设景观大道，打造蔷薇河、东盐河、玉带河等滨水生态廊道，做美城市空间。坚持集约化、系统化，复合提升城市功能，推进城市健康成长，形成生产、生活、生态协调统一的城市生命体。

建设活力动感城市。加快建设铁人三项等运动基地，推动建设游艇赛艇码头。高水平规划建设跨海索道，探索开发连岛至花果山直升机观光项目，建设一流的滑翔伞基地和独具特色的环云台山自行车道。大力发展水上运动、低空飞行、房车露营等业态，完善“10分钟体育健身圈”，增加健身站点，积极开展全民健身活动，常年参加体育锻炼人口达38%。举办徐圩国际马拉松、花果山国际越野挑战赛、首届“一带一路”国际铁人三项等有影响的体育赛事，塑造特色鲜明的城市性格，形成活力迸发的城市精气神。

建设宜居品质城市。统筹推进文明、生态、智慧、健康连云港建设。完善功能配套，补齐设施短板，合理布局生活、娱乐、购物设施，为市民提供触手可及的便利。实施华联广场、解放东路片区等27个棚改项目，推进沿街亮化、立面整治，全面消除城市无灯区，推进多层住宅加装电梯，提高市民生活舒适度。实施53项市政项目，扎实推进海绵城市、地下管廊等建设，加强水电气供应和市政设施配套，加快打通“断头路”，畅通城市血脉经络。加快5G试点部署，推进数据共享，完善城市管理信息系统，以智能化让城市变得“耳聪目明”。深入开展文明城市创建，组织实施“八大工程”，弘扬社会主义核心价值观，提升市民科学文化素养，推进公共服务体系建设。大力倡导全民阅读，以满城书香涵养城市，让城市由内而外散发出文明气质。

（五）推进乡村振兴战略，加快城乡融合发展。深入实施“十项工程”，抓重点、补短板、强弱项，引导更多要素向农村流动，完善基础设施和公共服务，让农业有奔头、农民有盼头、田园乡村有看头。

发展现代农业。推进农业产业化，稳定粮食生产，壮大优势产业，创建国家级农村特色产业融合示范园1个，新增市级以上产业化联合体20个、农业龙头企业40家，新增观光农业示范村（点）60个。提升农业品牌化，突出优质、特色、绿色，加快结构调整，“三品一标”农产品达40%以上。创建农产品质量安全市，放大“连天下”品牌效应。农村电商销售增长50%以上。加快农业外向化，推进国家农业对外开放合作试验区建设，农产品出口保持全省前列。促进农业科技化，强化与科研院所合作，加快成果转化，农业科技进步贡献率达66%。大力推进农业基础设施现代化，推进土地整理项目，新建高标准农田21万亩，新增高效设施农业3万亩，农业机械化综合水平达84%、信息化率达59%。

推进新型城镇化。稳步提升城镇发展水平，构建“一横两纵、一核多极”城镇格局。加快县城中等城市建设，提高综合承载功能和公共服务能力，促进县城规模适度扩张。扎实推进重点中心镇建设，建成一批特色鲜明、独具格调的“小城”样板。依托产业特点、人文底蕴和生态禀赋，坚持特色化培育、差异化发展，打造一批国家级、省级特色小镇。高起点规划建设空港小镇、水晶小镇等产业小镇，促进区域发展转型升级。

改善农村人居环境。因地制宜推进村庄搬迁撤并，试点建设47个新型社区，引导农民进城入镇，加快改善农民群众住房条件。推进特色田园乡村建设，新增3个以上省级试点村庄。做好垃圾污水处理、厕所革命、村容村貌提升等工作。整治交通干线沿线环境、实施镇村道路提升行动，完成剩余650公里农村公路改造，镇村公交覆盖率90%以上。健全乡村治理体系，弘扬优秀乡土文化，推动形成文明乡风、良好家风、淳朴民风。

（六）坚决打好三大攻坚战，加快补齐发展短板。三大攻坚战，事关经济发展质量和全面小康分量。我们须以更加坚定的决心，更加务实的举措，坚决打赢打好。

打好防范化解重大风险攻坚战。坚持结构性去杠杆，有效防控金融风险，严厉打击非法集资、违规融资担保，推进互联网金融风险专项整治。完善地方金融监管体制，强化农民资金互助社运营监管，确保不发生区域性系统性风险。加强政府债务管理，化解存量，控制增量，做到坚定、可控、有序、适度。加强信息研判，排查化解矛盾，确保不发生有影响的重大事件。

打好精准脱贫攻坚战。对照国家脱贫标准和“两不愁三保障”要求，保持工作力度不减、帮扶标准不降，切实抓好“六个精准”。强化就业扶贫，让更多农民实现家门口就业。大力推进产业扶贫，省定经济薄弱村全部建有富民项目。深化行业扶贫，建立农村义务教育辍学防控机制，实施低收入人口大病专项救助，加快贫困片区道路、饮用水等设施建设。更加突出兜底保障，实现剩余贫困人口和省定薄弱村全部脱贫，确保不返贫。

打好污染防治攻坚战。坚持把化工园区彻底整治作为生态优先、绿色发展的重要拐点，坚决打赢生态保护持久战。深入抓好中央环保督察“回头看”反馈问题整改和“263”专项整治，切实提升污染物收集、处置、清洁能源利用及环境监测监控“四个能力”。深化“蓝天”行动，坚持联防联治、综合治理，统筹推进控煤、管车、治气、降尘、禁烧等工作，完成“散乱污”企业综合整治，4家钢铁厂全部实施超低排放改造，空气优良率保持全省领先。实施“碧水”行动，巩固整治成果，深化“一河一策”，国省考断面及入海河流全面消除劣Ⅴ类，城区黑臭水体全面消除，49个工业集聚区污水全部实现集中处理达标排放。加快镇村污水处理设施建设，规模化畜禽养殖场治理率达95%以上。开展“净土”行动，对530家重点行业企业土壤污染状况进行调查，稳步推进德邦原址等地块土壤治理，固危废规范化管理达标率80%以上。加强山体绿化、宕口修复，加大湿地、林地保护力度，严控各类开发活动，严守生态保护红线。

各位代表，生态环境是最宝贵的财富，必须像保护眼睛一样精心呵护。我们将坚持政府主要负责人直管环保的机制不变，力度不减，“火眼金睛”强化监管，把山水林田湖等生态资源守护好、利用好，让蓝天碧海、绿水青山成为港城最靓丽的名片。

（七）坚持以人民为中心，加快提升民生福祉。人民对美好生活的向往，就是我们的奋斗目标。我们将用心用情多办实事，加快发展民生事业，努力让群众有更多获得感、幸福感、安全感。

大力促进就业创业。实施积极就业政策，完善服务体系，优化就业结构，提高就业质量，统筹做好城乡困难人员、返乡农民等群体就业帮扶。新增城镇就业6万人，城镇失业人员再就业1.6万人，转移农村劳动力2.5万人，城镇零就业家庭、农村零转移家庭动态清零。建设一批实训见习基地，开展技能培训25万人次。扎实推进“创响港城”计划，落实创业政策“28条”，丰富创业形式，增加创业载体，重点扶持大学生等万人以上自主创业，带动就业4万人。鼓励支持各类人才返乡创业、下乡兴业，拓展农村就业空间和农民增收渠道。弘扬劳模精神和工匠精神，营造劳动光荣的社会风尚和精益求精的敬业风气。

健全公共服务体系。以“四个一流”标准，优先发展教育，新改扩建幼儿园、中小学36所，清理整顿无证幼儿园，规范校外培训。城区小学全面推行“弹性离校”，扩大午餐供应试点。增加高中学位供给。特教学校实现搬迁。提升中高职院校办学水平，加快创建江苏海洋大学，支持康达学院改善办学条件。大力发展民办教育。提高医疗服务水平，市妇幼保健中心新院区投用，加快市二院、中医院、康复医院等重点工程建设。建成国家级卫生乡镇20个。深化公立医院改革，推进“互联网+医疗”服务，加强全科医生培养，深入推进“健康宝贝工程”。坚持守正创新，繁荣文化事业，实施文脉整理工程，推进历史文化名城申建，推出淮海戏《孤岛夫妻哨》等精品力作，组织万场电影千场戏展映展演。创新举办载体，办好连云港之夏、西游记文化节、首届“一带一路”国际音乐节。

完善社会保障体系。实施全民参保计划，提升城乡居民基本医疗保险报销比例，提高养老保险基础养老金，保障被征地农民社保权益，对因病致贫对象实施医疗救助，对困难人员灵活就业发放社会保险补贴。完善社会救助、社会福利、慈善事业等制度，健全城乡居民低保、农村“五保”、城市“三无老人”和孤儿供养标准自然增长机制，关爱困境儿童、留守儿童。做好残疾人康复、托养服务。新建标准化居家养老服务中心30个，护理型床位占比45%以上。推动住房公积金归集扩面，分配各类保障性住房不低于1000套，住房租赁补贴惠及更多城镇困难家庭。加强退役军人和优抚对象服务保障，促进军民融合发展，争创全国双拥模范城“八连冠”。

加强社会治理创新。深入推进“最安全城市”创建，建立现代化社会治安防控体系。扎实开展“七五”普法，深化执法司法规范化建设。深入推进信访矛盾化解，确保信访总量持续下降。加强社区综合服务中心建设，深化“三社联动”和“一委三会”治理。完善社会信用体系。对不良房地产开发等企业实施惩戒，建立淘汰曝光机制。强化食品药品监管，保障群众舌尖上的安全。完善应急管理体制机制，做到警钟长鸣，坚决防范重特大事故发生。进一步做好民族宗教、外事侨务、地震、民防、气象、邮政、科普、档案、史志、关心下一代、老促会、老科协、红十字、援疆等工作。

各位代表，民心是最大的政治，办好民生实事是政府最大的成绩。我们坚持开门办民生，面向社会广泛征集意见建议，开通了“两代表一委员”专门通道，聚焦群众需求，采取“百姓点菜”和“政府配餐”相结合，排定50件民生实事。我们将全力以赴，善作善成，真正将实事办到百姓的家门口，群众的心坎上。

全面加强政府自身建设

打铁还需自身硬，实现“高质发展、后发先至”，必须加强政府自身建设，切实提升施政能力和服务水平，更好履职尽责，不负人民重托。

（一）旗帜鲜明讲政治。自觉以习近平新时代中国特色社会主义思想武装头脑、指导实践，强化“四个意识”，做到“两个维护”。对中央大政方针、省委省政府和市委决策部署，坚决贯彻落实，绝不走样，绝不变通，绝不打折扣。深刻认识我市在国家发展大局中的定位，全面把握“一带一路”强支点、标杆示范、东中西区域合作等丰富内涵，提高发展站位，增强战略定力，更加自信自觉肩负起时代赋予连云港的重大使命。

（二）务实高效优服务。结合机构改革，调整优化职能，使职责更加清晰，运转更加高效。倡导雷厉风行、急事急办、特事特办，做到立说立行、马上就办，切实提高政府执行力。始终心系群众，推动重心下移，面对面、心贴心、实打实地服务群众、服务基层。坚持问题导向，发挥市长信箱、12345在线平台作用，积极解决群众反映问题，对群众合理诉求绝不说“不”。

（三）勇于创新敢担当。弘扬创新精神，用创新思维谋划工作，用改革办法破解难题，想他人没有想到的招数，干他人想干而没有干成的事情。强化“四实四干”，主动担当作为，坚决整治遇事“踢皮球”、为了不出事宁可不干事等消极懈怠行为，勇挑最重担子，善接最烫山芋，敢啃最硬骨头。落实“三个区分开来”，健全“三项机制”，为创新者鼓劲，为改革者撑腰，为担当者担当。

（四）依法行政严规范。严格执行“三重一大”决策制度，政府重大决策出台前向人大及其常委会报告，邀请人大代表、政协委员和市民代表列席政府常务会议。自觉接受人大法律监督、工作监督和政协民主监督，高度重视社会和舆论监督，高质量办理人大代表议案建议和政协提案，广泛听取民主党派、工商联、无党派人士意见。加强行政执法监督。推进数字政府建设，整合发展政务新媒体，健全发布、解读、回应“三位一体”政务公开体系。

（五）永葆清廉守规矩。加强廉洁政府建设，推进审计监督全覆盖，强化重点领域、关键环节、重要岗位硬约束。常念“紧箍咒”，持续整“四风”，紧盯不敬畏、不在乎、喊口号、装样子问题，绝不允许表态好、调门高、落实差、搞“忽悠”。严格落实中央“八项规定”精神。加强纪律教育，让广大公务人员存戒惧、讲规矩、守底线，树立勤政廉政的良好形象。

各位代表，“高质发展、后发先至”的光荣使命重任在肩，530万港城人民对美好生活的向往催人奋进，让我们紧密团结在以习近平同志为核心的党中央周围，在中共连云港市委的坚强领导下，勇毅笃行，锐意进取，以更加优异的成绩庆祝中华人民共和国成立70周年！
